# The Lausanne Infant Crying Stress Paradigm: Validation of an Early Postpartum Stress Paradigm with Women at Low vs. High Risk of Childbirth-Related Posttraumatic Stress Disorder

**DOI:** 10.3390/jpm11060472

**Published:** 2021-05-26

**Authors:** Vania Sandoz, Suzannah Stuijfzand, Alain Lacroix, Camille Deforges, Magali Quillet Diop, Ulrike Ehlert, Marius Rubo, Nadine Messerli-Bürgy, Antje Horsch

**Affiliations:** 1 Institute of Higher Education and Research in Healthcare (IUFRS), University of Lausanne, 1010 Lausanne, Switzerland; Vania.Sandoz@bluewin.ch (V.S.); Suzannah.Stuijfzand@protonmail.com (S.S.); Alain.Lacroix@chuv.ch (A.L.); Camille-Deforges@hotmail.fr (C.D.); Magali.QuilletDiop@gmail.com (M.Q.D.); 2 Department of Clinical Psychology and Psychotherapy, University of Zurich, 8050 Zurich, Switzerland; U.Ehlert@psychologie.uzh.ch; 3 Clinical Child Psychology & Biological Psychology, University of Fribourg, 1701 Fribourg, Switzerland; Marius.Rubo@unifr.ch (M.R.); Nadine.Messerli@unifr.ch (N.M.-B.); 4 Neonatology Service, Department Woman-Mother-Child, Lausanne University Hospital and University of Lausanne, 1011 Lausanne, Switzerland

**Keywords:** PTSD, cortisol, heart rate variability, stress reactivity, childbirth, TSST, postpartum, women, mothers, perceived stress

## Abstract

Stress reactivity is typically investigated in laboratory settings, which is inadequate for mothers in maternity settings. This study aimed at validating the Lausanne Infant Crying Stress Paradigm (LICSP) as a new psychosocial stress paradigm eliciting psychophysiological stress reactivity in early postpartum mothers (*n* = 52) and to compare stress reactivity in women at low (*n* = 28) vs. high risk (*n* = 24) of childbirth-related posttraumatic stress disorder (CB-PTSD). Stress reactivity was assessed at pre-, peri-, and post-stress levels through salivary cortisol, heart rate variability (high-frequency (HF) power, low-frequency (LF) power, and LF/HF ratio), and perceived stress via a visual analog scale. Significant time effects were observed for all stress reactivity outcomes in the total sample (all *p* < 0.01). When adjusting for perceived life threat for the infant during childbirth, high-risk mothers reported higher perceived stress (*p* < 0.001, *d* = 0.91) and had lower salivary cortisol release (*p* = 0.023, *d* = 0.53), lower LF/HF ratio (*p* < 0.001, *d* = 0.93), and marginally higher HF power (*p* = 0.07, *d* = 0.53) than low-risk women. In conclusion, the LICSP induces subjective stress and autonomic nervous system (ANS) reactivity in maternity settings. High-risk mothers showed higher perceived stress and altered ANS and hypothalamic–pituitary–adrenal reactivity when adjusting for infant life threat. Ultimately, the LICSP could stimulate (CB-)PTSD research.

## 1. Introduction

### 1.1. Childbirth-Related Posttraumatic Stress Disorder

Posttraumatic stress disorder (PTSD) may develop following a traumatic event, as defined by the PTSD stressor criterion of the Diagnostic and Statistical Manual of Mental Disorders, 5th ed. (DSM-5) [1]. Four symptom clusters (criteria B–E of the DSM-5) characterize this disorder: intrusions, avoidance of trauma-related cues, negative cognitions and mood, and hyperarousal [1]. PTSD can be diagnosed one month following the traumatic stressor [1], even if an acute posttraumatic stress response can be observed in the meantime [2]. The question arises to understand why after having been exposed to a traumatic event, only some individuals develop PTSD symptoms [3]. According to psychobiological findings, altered stress reactivity, such as a dysregulation of the hypothalamic–pituitary–adrenal (HPA) axis or the autonomic nervous system (ANS), may play a major role in the development of PTSD [4,5].

Up to one-third of mothers report perceiving giving birth as traumatic [6,7,8] and may develop childbirth-related PTSD (CB-PTSD) [9,10]. According to the DSM-5 PTSD stressor criterion, perceived life threat is a key element contributing to the traumatic appraisal of childbirth [1]. Therefore, mothers who fear for their life or physical integrity, or the life or physical integrity of their infant, during childbirth are at higher risk of CB-PTSD compared to those who do not [1,11]. In community samples, 3–4% of women reach the clinical threshold for CB-PTSD [12,13], whereas in high-risk samples, CB-PTSD prevalence rates increase up to 16–19% [12,13]. Studies investigating the relationships between CB-PTSD and traumatic childbirth experience and parity reported inconsistent results [14,15,16,17]. Although traumatic childbirth perception depends on the maternal subjective appraisal of the event, objective elements, such as obstetric or neonatal complications, can worsen the traumatic experience [11].

There are several arguments to assume that CB-PTSD might differ from PTSD by the nature of its trauma [18,19]. As a positive and expected life event, childbirth is generally perceived positively by society. Mothers might be terrified not only for themselves but also for their infant, which differs from other traumatic events. The perceived life threat for the infant is a strong predictor of CB-PTSD [17]. Accordingly, mechanisms involved in the development of CB-PTSD might be different from the ones of PTSD following other traumatic events.

Besides the maternal distress and interference with everyday functioning due to CB-PTSD symptoms [9], CB-PTSD can negatively affect the couple relationship [20,21] and subsequent future reproductive experiences [22,23]. Maternal CB-PTSD symptoms may also be associated with child sleep problems [22,24], breastfeeding initiation and continuation [22,25], and, potentially, with problems regarding the mother–infant relationship or bonding [22,26].

To date, evidence-based interventions to prevent CB-PTSD are lacking [27]. Given the significant consequences of CB-PTSD on mothers and their families, it is fundamental to better understand the underlying physiological mechanisms that play a role in the development of CB-PTSD, as this may open up options for the early assessment and treatment of CB-PTSD [28].

### 1.2. Psychophysiological Stress Reactivity and Posttraumatic Stress Disorder

The stress response system is composed of the sympathetic (SNS) and the parasympathetic (PNS) branches of the ANS and the HPA axis [29]. Under stress conditions, HPA activation increases (elevation of cortisol release), whereas PNS activation withdraws (reduction in high-frequency (HF) power), and SNS activation intensifies (increase in low-frequency (LF) power), resulting in a short-term imbalance of the ANS (assessed by LF/HF ratio) [29]. Cortisol release peaks at 10 to 30 min after stress exposure, whereas changes in SNS and PNS activation are seen more immediately [29]. Cortisol is released to mobilize physiological and psychological resources of the organism and to support recovery by counteracting the physiological effects of the SNS activation [29]. Cortisol release gradually increases during pregnancy, with a peak at the end of the pregnancy, and then returns to nongravid levels 12–24 h after childbirth [30,31,32,33]. There is evidence that early postpartum conditions can have an additional impact on cortisol release, such as breastfeeding within the last hour that causes blunted cortisol stress reactivity [34].

Overall, the literature suggests that the development and maintenance of PTSD are related to HPA dysregulation [35]. Individuals exposed to trauma or with PTSD show mainly blunted stress responses [35,36,37,38], and only one study with childhood abuse-related PTSD in women showed elevated salivary cortisol responses following exposure to traumatic reminders compared to women with a history of childhood abuse but without PTSD [39]. However, given the small sample size (*n* = 12 per group) and the unstandardized stress procedure, the interpretation of these findings requires caution. All other studies reported blunted cortisol reactivity in response to laboratory stress induction (e.g., Trier Social Stress Test (TSST), Emotional Stroop task), including a study on US female veterans compared to civilian women, regardless of their PTSD status [38], or in postpartum women with emotion regulation difficulties and a history of child maltreatment [37]. Similar results were found in mothers suffering from interpersonal violence-related PTSD at 12 and 48 months postpartum [40].

PTSD patients had altered ANS responses at rest compared to controls in a meta-analysis (i.e., reduced HF and LF power, with a higher reduction in HF than LF, and increased LF/HF ratio), indicating that the cardiovascular system cannot properly adapt to external and internal demands [5]. Further, in response to lab-based stress tasks, individuals showed an increase in SNS, a decrease in PNS activation, and a short-term imbalance of the ANS [41,42,43]. As far as we know, there is no study that investigated early postpartum mothers with a traumatic childbirth experience who might be at risk of CB-PTSD, even though hormonal changes linked to childbirth might influence ANS and HPA activation. It furthermore remains unclear whether the physiological changes can already be expected early after trauma at a time point where PTSD is not yet established. In addition, stress also provokes a psychological stress response, such as perceived stress [44,45]. To our knowledge, no study has yet investigated early perceived stress reactivity in women following traumatic childbirth or in individuals at risk of CB-PTSD.

### 1.3. The Current Study

Assessment of stress responses during the early postpartum period is limited due to the physical and psychological constraints of the early postpartum period in women (e.g., standing up during the stress phase can be physically impossible, and pretending to be in a job interview, as it is used in standard lab-based stress tasks, is likely to be far from maternal concerns during the days following childbirth) [46,47,48]. Nevertheless, studying psychophysiological stress reactivity in mothers during the early postpartum period could be relevant to identify CB-PTSD risk factors and to clarify physiological mechanisms underlying the development of CB-PTSD [35].

Therefore, the current study firstly aimed to provide the first evidence for the validation of a new stress paradigm, the Lausanne Infant Crying Stress Paradigm (LICSP), for the early postpartum period. More specifically, we hypothesized a psychophysiological stress response to the LICSP (i.e., increase in salivary cortisol release, reduction in HF power, increase in LF power, increase in LF/HF ratio, and increase in perceived stress).

Given that the general PTSD literature reported altered physiological stress reactivity in traumatized individuals, this study secondly intended to investigate group differences in psychophysiological stress reactivity in women at low vs. high risk of CB-PTSD in the early days following childbirth. However, given that stress reactivity has never been studied shortly after the traumatic stress exposure, exploratory analyses were conducted for group differences.

## 2. Materials and Methods

### 2.1. Design and Study Population

This cross-sectional experimental study took place in the postpartum ward of the maternity unit of a Swiss university hospital between December 2018 and December 2019. The following inclusion criteria were applied: (a) being ≥ 18 years old, (b) having given birth to a live baby at ≥34 weeks of gestation during the previous ≤5 days, (c) having given written consent, and (d) obtaining a certain score in response to screening questions that allowed the allocation to the groups at low vs. high risk of CB-PTSD. For this purpose, willing mothers answered screening questions consisting of two items related to perceived life threat during childbirth (based on PTSD stressor criteria of DSM-5) [1,18,48]. Responses were scored on a 7-point Likert scale (1 = not at all to 7 = extremely): “Did you think that your life was in danger?” “Did you think that your baby’s life was in danger?” To be included in the study, low-risk participants had to respond ≤2 to each of the two items, whereas high-risk participants had to score ≥4 on one of the two items. The reason for such classification was to ensure participants were either not traumatized or sufficiently traumatized to find group differences. Exclusion criteria included (a) insufficient French language skills, (b) established intellectual disability or psychotic illness, (c) antenatal corticosteroid administration, (d) current alcohol abuse and/or illegal drug use, (e) severe maternal and/or infant illness, and (f) infant hospitalized in neonatal intensive care unit. The total sample consisted of 52 participants, with 28 mothers in the low-risk group and 24 in the high-risk group.

As part of the Swiss Traumatic Birth Trial (START) [18], this study was conducted in accordance with the Declaration of Helsinki and was approved by the local ethics committee for research in humans (study number 2017-02142). Before enrolling in the current study, participants received an information sheet and gave written consent. The current study was written up according to the STROBE reporting statements [49].

### 2.2. Procedure and Measures

#### 2.2.1. The Lausanne Infant Crying Paradigm Procedure

The LICSP development was based on the principal components of the TSST, which is the gold standard paradigm to induce physiological stress responses in adults [46,47,48]. The LICSP procedure comprised a baseline phase, a stress phase, and a recovery phase, ending with an 8 min optional guided relaxation. Both the baseline and the recovery phases lasted 40 min, whereas the stress phase lasted 20 min.

Mothers were encouraged to breastfeed and were asked to have lunch before starting the LICSP at 1 p.m. Visitors were prohibited during the whole LICSP procedure, which coincided with the time the maternity did not allow other visitors than partners (outside of public visiting hours). During the baseline and the recovery phases, participants were instructed to rest in their hospital bed with their baby around and their partner (if they were present). During the stress phase, participants were alone in a separate consultation room sitting at a desk and facing an unknown female experimenter that observed them, gave them instructions, and adopted a neutral facial attitude [47]. All participants were filmed on the pretext that their facial expressions and their stress coping skills would be analyzed by specialists. The stress phase consisted of a 3 min anticipation of the infant crying test (ICT), the ICT being a psychosocial stressor, and a surprise mental arithmetic task as a cognitive stressor [47]. The ICT involved differentiating one’s own infant crying among several recordings of infant crying. The experimenter read instructions implying that mothers usually differentiate their infant’s crying from those of others, which can threaten the social self of participants and induce stress [46,50]. Actually, mothers can recognize their own infant crying already at 24 h postpartum, but they cannot differentiate it from the crying of other infants [51]. The instructions of the surprise mental arithmetic task did not differ from the ones used in the TSST [47].

The ICT was designed and run with E-Prime 3.0 software (Psychology Software Tools, Pittsburgh, PA, USA). Five 20 s recordings of infant crying were first played to participants through headphones, followed by 14 other 10 s infant crying recordings. Between each infant crying recording, participants had 3 s to indicate by pressing on specific keyboard keys whether this was their own infant crying or not. Among the 19 infant crying clips, one of 10 s length and another of 20 s were from the participant’s own infant. Those two crying clips were recorded prior to the LICSP during a routine care episode (e.g., diaper change, bath) in the absence of the participant.

**Figure 1:**
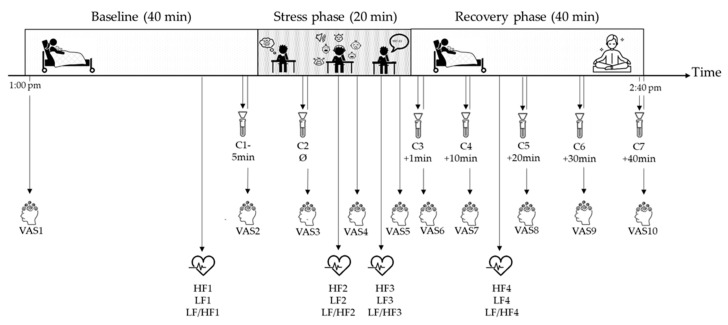
The procedure of the Lausanne Infant Crying Stress Paradigm contains a baseline phase and recovery phase, in which participants rest in their hospital room with their baby, as well as a stress phase, in which participants complete three stressful tasks (i.e., 3 min anticipation of the infant crying test, the infant crying test, and a surprise mental arithmetic task) in a consultation room. At the end of the recovery phase, an optional guided relaxation is offered to participants. Salivary cortisol assessments are represented by salivette icons, with the 1st salivary sample (C1) taken 5 min before the beginning of the stress phase, the 2nd salivary sample (C2) taken after the 3 min anticipation before the psychosocial and cognitive stressors, and the 3rd to 7th salivary samples (C3–C7) taken after the stress phase at 10 min interval. Perceived stress is illustrated with the head icon, measured 10 times via a visual analog scale (VAS) ranging from 1 = not at all stressed to 5 = extremely stressed. Heart rate variability covering high-frequency (HF) power, low-frequency (LF) power, and LF/HF ratio is symbolized by the heart icon and is measured at four different time points.

Both the psychosocial and cognitive stressors contained the main evidence-based characteristics required to elicit psychosocial stress: anticipation, novelty and/or unpredictability, social-evaluative threat, uncontrollability, and motivated performances [45,46,52]. Figure 1 illustrates the LICSP procedure, including time points of measurements. At the end of the recovery phase, a guided relaxation was offered to participants, followed by a moment to debrief to ensure no side effects would result from the stress phase. Finally, participants completed a set of questionnaires (see Section 2.2.3) right after the LICSP or during their stay in the postpartum ward.

#### 2.2.2. Psychophysiological Stress Responses

Given that saliva collection does not induce additional stress and is noninvasive and reliable, salivary cortisol was chosen as a measure of the HPA activity in response to stress [52,53]. As illustrated in Figure 1, saliva was obtained using Salivettes (Sarstedt, Sevelen, Germany) 5 min before the start of the stress phase (C1) and directly after the 3 min anticipation of the ICT (C2). Five additional samples were collected during the recovery phase at 10 min intervals (after the stressor; during early, mid, and late recovery; and at the end of the paradigm (C3 to C7)). The timing of salivary collection is consistent with the standardized protocol of the TSST, with a salivary cortisol peak response expected between 10 and 30 min post-stress (i.e., C4 to C6) [29,47]. Participants were instructed not to eat, drink, chew gum, or brush teeth within 30 min before saliva collection. After chewing the cotton wool for 60 s, salivary samples were stored at ≤20 °C until they were sent for analysis to the biochemical laboratory of the Clinical Psychology and Psychotherapy Department at the University of Zurich, Switzerland. Cortisol levels (nmol/L) were analyzed using luminescence immunoassay based on the competition principle (IBL, Hamburg, Germany). Inter- and intra-assay coefficients of variance of cortisol were ≤5%.

Cardiac activity was measured continuously via a Firstbeat Bodyguard 2 (Firstbeat Technologies Ltd., Jyväskylä, Finland) ECG device. During the 3 min recordings of the baseline (i.e., HF1, LF1, and LF/HF1) and recovery (i.e., HF4, LF4, and LF/HF4) phases, participants were instructed to rest in their hospital beds and were allowed to be with their baby. The Kubios HRV Standard software (ver. 3.2.0) was used to graphically display the cardiac activity in order to spot outliers. Data quality was assessed by inspecting the normality of interbeat intervals (IBI values) for each recording and by comparing HRV metrics with and without the Kubios’ artifact correction algorithm applied (set to medium). Data quality was high in all recordings; therefore, no artifact correction was necessary. Frequency-domain HRV parameters (i.e., HF power, LF power, and LF/HF ratio) were calculated using fast Fourier transformation. LF power (range: 0.04–0.15 Hz) reflects mainly SNS activation, whereas the HF power (range: 0.15–0.4 Hz) echoes the PNS activation [41,43]. The LF/HF ratio illustrates the balance of SNS and PNS activity [41,43].

Perceived stress was measured 10 times via a visual analog scale (VAS) rating from 1 = not at all stressed to 5 = extremely stressed (Figure 1) [54]. Perceived stress was assessed at the beginning and the end of the baseline phase, during the stress phase, at the end of the anticipation period and of the psychosocial and cognitive stressors, and, finally, during recovery at each time point of saliva sampling.

#### 2.2.3. Psychosocial and Medical Information

Perceived life threat for the mother and the infant during childbirth was measured via the screening questions (see Section 2.1). To situate the sample and given that depression is frequent and comorbid with acute stress during the early postpartum period [55], depression was assessed via the Edinburgh Postnatal Depression Scale (EPDS) [56]. This self-report questionnaire assesses the severity of postnatal depression symptoms over the past week [56]. A sentence was added to instructions stating that researchers were particularly interested in how mothers feel since childbirth. The 10 items are scored on a 4-point Likert scale (0–3), with a total score ranging from 0 to 30 [56]. A higher score suggests a higher level of symptom severity [56]. The French version has demonstrated satisfying psychometric proprieties [57]. The internal consistency observed in the present study was good (Cronbach’s α = 0.85) and was slightly higher than that previously reported in postpartum mothers of Switzerland [58,59].

Since anxiety is also common in the early postpartum period and comorbid with acute stress [55], anxiety was measured through the anxiety subscale of the Hospital Anxiety and Depression Scale (HADS-A). This 7-item self-report questionnaire retrospectively measures the severity of anxiety symptoms within the week preceding childbirth, using a 4-point Likert (0–3) scale that ranges from 0 to 21 [60]. Higher scores indicate greater symptom severity [60]. The French version has shown good psychometric characteristics [61] with Cronbach’s alpha of 0.72, which is similar to previous studies of Swiss postpartum women [55,62].

Participants responded to psycho-sociodemographic questions on age, civil status, education level, and smoking habits. Parity, gravidity, and type of delivery were retrieved from medical records. In addition, a dichotomous variable was created depending on whether the participant breastfed over the 60 min preceding the stress phase (*yes* = 1, *no* = 2).

### 2.3. Evaluation of the Stress Phase of the Lausanne Infant Crying Stress Paradigm

After the LICSP and as a manipulation check, participants were asked to evaluate the stress phase with a 100-point Likert scale (0 = not at all, 50 = moderately, 100 = extremely) according to the following parameters: novelty, difficulty, stress, controllability, and predictability [46,63].

### 2.4. Sample Size Calculation

Given that the LICSP is a new stress paradigm, no prior data existed for an a priori power calculation. Therefore, a sample size of *N* = 40 was estimated based on previous studies using the TSST (i.e., the gold standard stress paradigm) [37,46,64]. The current study consequently aimed to include 50 mothers completing the LICSP procedure to account for a potential lower effect of the LICSP.

### 2.5. Data Analysis

All the statistical analyses were carried out using R v3.6.1 (running under RStudio v1.1.463) [64]. The salivary cortisol levels of two participants were missing for C1, and the perceived stress ratings of one participant were missing for the last assessment (VAS10). Given that these missing data represented <30% of the dataset, they were imputed using Bayesian linear regression (NORM) for numerical values. The missing data imputation was performed by the mice package v3.11.0 algorithms [65]. Due to technical issues, the cardiac activity of one participant during the stress phase was missing. Given that more than 30% of her data were missing, these data were not imputed. Further, seven participants did not entirely perform the mental arithmetic task, but HRV data were not imputed.

Results were considered significant at *p* < 0.05. Descriptive differences between the low- and high-risk groups were assessed with the appropriate statistical tests, namely the chi-square test (*X*^2^), the Fischer’s exact test (*p*), or the Mann–Whitney test (W). Given that data did not meet assumptions for a two-way repeated-measures analysis of variance (ANOVA; group*time), the aligned ranks transformation repeated-measures ANOVA (ART ANOVA; group*time), i.e., a nonparametric test, was carried out. To assess stress reactivity to the LICSP, pairwise comparisons with Tukey adjustments were conducted as post hoc tests to determine differences between relevant pair groups. Effect sizes (Cohen’s d) were calculated. Finally, one-way analyses of covariance (ANCOVAs) were conducted to determine differences between low- and high-risk groups in psychophysiological stress responses, using perceived life threat for the infant as a covariate. Results are reported as unadjusted and adjusted for perceived life threat for the infant. Given the potential impact of breastfeeding on salivary cortisol activity, an additional exploratory ANCOVA was carried out for this outcome. Note that, given the inconsistent literature on CB-PTSD or traumatic childbirth experience and parity, parity was not considered as a covariate in the current paper [14,15,16,17]. Finally, cortisol levels were expected to be low at C1 and C2. However, as shown in Table 1, participants showed elevated cortisol release at C1. Therefore, C2 was considered as a baseline value for all the statistical analyses.

## 3. Results

### 3.1. Characteristics of the Sample and of the Lausanne Infant Crying Stress Paradigm

The study was presented to *n* = 195 mothers, of whom *n* = 72 agreed to answer the screening questions. The main reasons for declining participation were being tired, imminent return home, or being unavailable at the time of the LICSP. Of those who participated in the screening process, *n* = 20 had a screening score that did not allow their group allocation; i.e., the perceived life threat of these women was not sufficiently low or high for them to continue with the study. The final study sample included *n* = 24 mothers at high risk of CB-PTSD and *n* = 28 CB-PTSD low-risk mothers. Two participants from the low-risk condition stopped the experiment after the stress phase and did not complete the recovery period.

Sociodemographic, medical, and psychological characteristics of the sample are presented in Table 2. The high-risk group reported a greater perceived life threat for the mother (*p* = 0.002) and for the infant (*p* < 0.001). The low- and high-risk groups significantly differed in the type of delivery, with more vaginal births and planned cesarian sections for the former and more operative vaginal births and emergency cesarian sections for the latter (*p* < 0.001). Further, low-risk mothers had a significant higher parity (*p* = 0.022) and gravidity (*p* = 0.013) than high-risk mothers. As illustrated in Table 3, no significant group difference was observed regarding procedural characteristics of the LICSP. On average, the baseline phase lasted 44 min (*SD* = 3 min), the stress phase lasted 22 min (*SD* = 3 min), and the recovery phase lasted 43 min (*SD* = 4 min).

### 3.2. Salivary Cortisol Response to Psychosocial Stress

**Figure 2:**
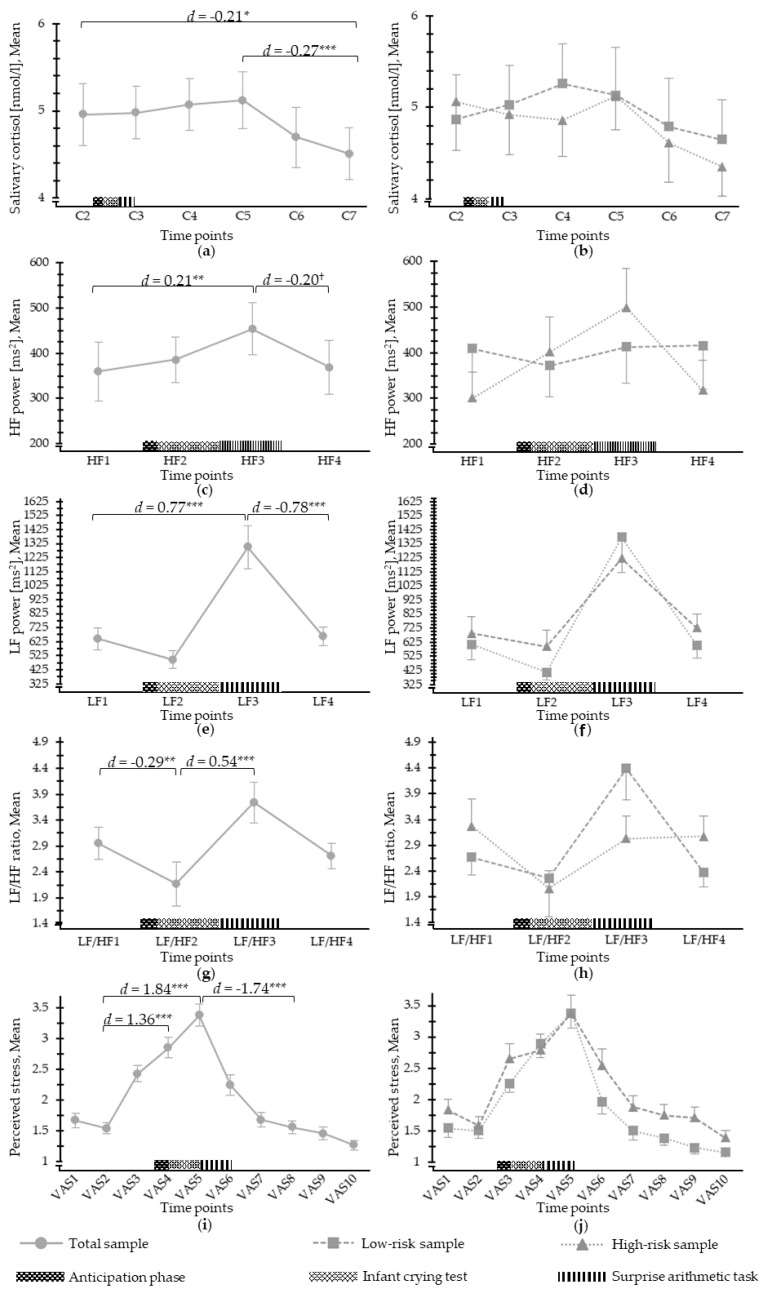
Mean psychophysiological stress reactivity during the different phases of the Lausanne Infant Crying Stress Paradigm: (**a**) mean salivary cortisol for the total sample; (**b**) mean salivary cortisol for the low- and high-risk groups; (**c**) mean HF power (HF, range: 0.15–0.4 ms^2^) for the total sample; (**d**) mean HF power for the low- and high-risk groups; (**e**) mean LF power (LF, range: 0.04–0.15 ms^2^) for the total sample; (**f**) mean LF power for the low- and high-risk groups; (**g**) mean LF/HF ratio for the total sample; (**h**) mean LF/HF ratio for the low- and high-risk groups; (**i**) mean perceived stress ratings (range: 1 = not at all to 5 = extremely) for the total sample; (**j**) mean perceived stress for the low- and high-risk groups. Error bars represent standard error. Graphs showing mean group stress response patterns over time are unadjusted and are displayed for illustrative purposes. When controlling for the perceived infant life threat, significant or marginal group effects on salivary cortisol release (*d* = 0.53 *), HF power (*d* = 0.53 ^†^), LF/HF ratio (*d* = 0.93 ***), and perceived stress (*d* = 0.91 ***) emerged that are not indicated in this figure. ^†^ *p* < 0.10, * *p* < 0.05, ** *p* < 0.01, *** *p* < 0.001.

Table 3 shows the characteristics of the salivary cortisol assessments during the LICSP. Analyses revealed a significant time effect (*F*(5, 250) = 4.84, *p* < 0.001) for salivary cortisol (Figure 2). Salivary cortisol increase between C2 (baseline) and C4 (expected peak response within early recovery period, 10 min post-stress) was not significant (*p* = 0.94, *d* = 0.01), but a significant difference was observed between baseline (C2) and recovery period C7 (end of paradigm, *p* = 0.027, *d* = −0.21) and within recovery at C5 (20 min post-stress) and C7 (end of paradigm; *p* = 0.003; *d* = −0.27), revealing that cortisol was high at baseline, kept the same level up to the expected peak time point, and was reduced afterward. There was a significant and moderate group effect when controlling for the perceived life threat for the infant (*F*(1, 309) = 5.20, *p* = 0.023, *d* = 0.53), with mothers of the high-risk condition showing slightly lower adjusted mean level of salivary cortisol (M = 4.26, SD = 1.96) than the ones of the low-risk group (M = 5.44, SD = 2.40). Further, an exploratory ANCOVA examining the role of breastfeeding showed no significant group effect on salivary cortisol levels.

### 3.3. ANS Response to Psychosocial Stress

ANS properties during the LICSP, including HF power, LF power, and LF/HF ratio, are displayed in Table 3. A significant time effect was observed for HF power (*F*(3;142.47) = 4.59, *p* = 0.006) (Figure 2), with a significant increase in HF power from the baseline (i.e., HF1) to the cognitive stressor (i.e., HF3) (*p* = 0.004, *d* = 0.21), followed by a HF power decrease during the recovery (i.e., HF4) (*p* = 0.066, *d* = −0.20). A moderate group effect was found for HF power when controlling for the perceived life threat for the infant (*F*(1;197) = 3.32, *p* = 0.07, *d* = −0.53). Mothers at risk of CB-PTSD showed higher adjusted mean of HF power (*M* = 508.01, *SD* = 351.36) than women at low risk (*M* = 287.79, *SD* = 469.47).

Regarding LF power, a significant time effect was observed (*F*(3;143.26) = 14.39, *p* < 0.001) (Figure 2). From the baseline (i.e., LF1) to the cognitive stressor (i.e., LF3), LF power significantly increased (*p* < 0.001, *d =* 0.77) before significantly decreasing during the recovery (i.e., LF4) (*p* < 0.001, *d* = −0.78), showing medium to large effect sizes. No significant group effect on mean LF power was detected, even when adjusting for the perceived life threat for the infant.

There was a mean LF/HF ratio time effect (*F*(3;143.47) = 10.42, *p* < 0.001) (Figure 2) with a decrease between baseline (LF/HF1) and the psychosocial stressor (i.e., LF/HF2, *p* = 0.002, *d* = −0.29) and an increase to the cognitive stressor (i.e., LF/HF3, *p* < 0.001, *d* = 0.54). The group effect for the mean LF/HF ratio was large and was only significant when controlling for the perceived life threat for the infant (*F*(1, 197) = 10.84, *p* < 0.001, *d* = 0.93), with mothers of the high-risk condition showing a lower adjusted mean LF/HF ratio (M = 1.59, SD = 2.16) than the ones of the low-risk group (*M* = 3.95, *SD* = 2.83).

### 3.4. Perceived Stress in Response to Psychosocial Stress

A significant time effect was reported for perceived stress (*F*(9; 450) = 43.10, *p* < 0.001) (Figure 2). Perceived stress from the baseline, i.e., VAS2, increased during the psychosocial and cognitive stressors (VAS4: *p* < 0.001, *d* = 1.36; VAS5: *p* < 0.001, *d* = 1.84) and then decreased during recovery, i.e., VAS8 (*p* < 0.001, *d* = −1.79). The group effect was large and significant when controlling for the perceived life threat for the infant (*F*(1, 517) = 25.89, *p* < 0.001, *d* = 0.91), but not otherwise. Hence, high-risk mothers reported more perceived stress (*M_adjusted_* = 2.55, *SD_adjusted_* = 1.17) than low-risk mothers during the LICSP (*M_adjusted_* = 1.53, *SD_adjusted_* = 1.07).

## 4. Discussion

Changes in psychophysiological stress responses occurring during the early postpartum period after traumatic childbirth have not been investigated so far. However, these changes might play a significant role in the development of CB-PTSD. In this study, the first evidence for the validation of a new stress paradigm, namely the LICSP, was collected regarding the ANS and subjective stress responses of mothers in the early postpartum period (2–3 days after childbirth).

Results revealed that the LICSP elicits ANS and subjective stress responses in women in the early postpartum period. Mothers perceived increased levels of stress and an increase in LF power, which corresponds with primary SNS activation as a response to the stress task. In parallel, HF power representing PNS activation also increased during the stress task, which did not correspond with our expectations. Further, salivary cortisol did not change in response to the stress task, but it maintained an increased level that already existed before the stress induction up to the expected peak time point in response to the stress task and declined afterward, showing significantly lower levels at the end of the paradigm than at baseline.

Further, the results of this study revealed higher perceived stress, higher HF power, and lower LF/HF ratio in the high-risk group when controlling for the role of the perceived life threat for the infant, with moderate to large effect sizes. Although these results need to be replicated, these group differences hint at future possibilities of identifying distinct early stress responses in mothers at high risk of developing CB-PTSD, with important implications for the early identification of those potentially in need of professional support.

In sum, the LICSP revealed stress responses to the new paradigm in the total sample of postpartum mothers. There was no increase in salivary cortisol in response to the LICSP, as mothers showed increased levels of cortisol already at baseline. Reasons for this could be diverse but are likely to be linked to the anticipation of participating in a study. First, mothers were instructed to breastfeed and have lunch before the start of the LICSP at 1 p.m. Managing these requirements, as well as the demands related to the early postpartum period (e.g., maternal and newborn care, hospital schedule for lunch) might have been a source of stress, augmenting the mean baseline salivary cortisol level. In addition, given that mothers knew they would be separated from their infant during the upcoming stress phase, they might have been more stressed by anticipating this moment. Therefore, assessment after the anticipation period (C2) showed lower cortisol levels than during the baseline (C1), and the cortisol levels remained higher than expected until after the expected peak in relation to the stress task. Potentially, an appropriate salivary cortisol baseline could have been obtained by prolonging the baseline period.

Overall, the mean cortisol levels observed in the current sample were higher than what was previously observed in traumatized vs. nontraumatized women [38]. In the early postpartum period, various confounders might have influenced HPA activation, including sleep deprivation and parenting challenges after birth [45,66,67,68]. Regarding the cortisol reactivity, estrogen, progesterone, oxytocin, and prolactin are known to play a major role in the initiation and maintaining of lactation and might blunt or reduce HPA activation during a stress task [69,70]. A study reported diminished blood cortisol reactivity in lactating women at 2 months postpartum [71]. In contrast, cortisol levels in our sample were increased. This discrepancy could be the result of the fact that assessment took place only a few days after childbirth when the lactation was only initiated, which is a different stage of the postpartum period and therefore difficult to compare. Further, cortisol levels could have been affected by hormonal conditions related to the previous pregnancy state, but there is evidence showing that maternal salivary cortisol should return to prepregnancy levels at 12–24 h postpartum [31,32].

Another reason for these results could be that the overall level of stress during the stress phase may not have been sufficiently intense to further activate the HPA axis [29]. The limited social-evaluative threat (i.e., when some part of the self is judged by others) might have caused less HPA activation during the LICSP [46], as, contrary to the stress phase of the TSST that contains a panel of experimenters [47], the stress phase of the LICSP includes only one experimenter. Therefore, the single experimenter might have had less of a social-evaluative impact, thus limiting the cortisol release. Further, the absence of face-to-face feedback from the experimenter on the participant’s performance during the ICT may have decreased the social-evaluative threat [72].

The results clearly showed an increase in perceived stress and LF power, a marker for SNS activation under stress, and an unexpected parallel increase in HF power. This is contrary to a previous study with pregnant women revealing HF power withdrawal, a decreased LF/HF ratio, but no change in LF power [42]. Given that following childbirth, the HPA activation changes back to nonpregnant stages, similar returns to a nonpregnant stage of ANS activation could have been expected [30,31,73]. However, an examination of the HF response pattern revealed an increase in HF power in the total sample and particularly in the high-risk group. This could be related to the pain condition after birth caused by compensatory sympathoadrenal activation that includes catecholamine release into the circulation system [74] in the total sample, which may result in parallel activation of SNS and PNS. It does not, however, explain the difference between the high- and low-risk groups. Interestingly, a previous study reported a similar increase in maternal PNS activation during a stress task that caused ruminations in relation to the threat of being separated from the child [73]. This coactivation of SNS and PNS in the high-risk group could be related to the levels of hypervigilance after the trauma [73] and could have caused contradictory PNS elevation during the task as mothers, while listening to infants’ crying, might have had ruminations linked to their baby’s wellbeing.

Moderate and large effects were found for differences between high- and low-risk groups in relation to other physiological stress responses. We, therefore, conclude that when controlling for the infant life threat, mothers at high risk of CB-PTSD report more perceived stress and show altered ANS and HPA activation during a stress task within the first days after childbirth. Psychophysiological stress mechanisms potentially involved in CB-PTSD development seem to be differently affected according to whether a danger was perceived for the mother or the infant during childbirth. This is not surprising given that admission to neonatal intensive care is a predictor of CB-PTSD [75]. This assumption is supported by recent evidence showing that PTSD and CB-PTSD differ in symptomatology [19,75,76,77]. Reassessment after a couple of weeks might reveal a different stress response pattern, particularly regarding the HPA response.

### 4.1. Strengths and Limitations

This study addresses a methodological gap in perinatal stress research. To our knowledge, this is the first study that examined physiological stress reactivity in mothers who recently gave birth using a stress paradigm, which allows assessment in a hospital ward context. The development of the LICSP was based on the gold standard of lab-based stress testing, the TSST, which is a robust stress paradigm [46,47]. The sample of this study included mothers at risk of CB-PTSD with lower parity and more operative vaginal deliveries and emergency cesarian sections, which are known to be risk factors for CB-PTSD [13,14,17], thus suggesting a good internal validity. The fact that the measurement of stress responses included psychological and physiological responses, namely both stress branches (HPA axis and ANS), and the application of a standardized procedure (LICSP) are strengths of the study. The manipulation check of the LICSP (Table 2) further revealed that high novelty, high difficulty, and low predictability but medium controllability were achieved.

Nonetheless, some limitations must be highlighted. First, the mean salivary cortisol level at baseline (C1) was higher than expected for a baseline [78]. Future research could address this by lengthening the baseline, although the circumstances in this early postpartum period might not allow this to be feasible. Second, early postpartum factors might have influenced stress responses and would need to be assessed in the future (e.g., oxytocin provoking a blunted HPA response) [69,70,71]. Third, sleep deprivation [66,67,68] or lifestyle factors (e.g., history of nicotine, alcohol, or caffeine use) [45] could have caused physiological changes, none of which were included here as covariates, given the small sample size. Fourth, potential PTSD symptoms resulting from previous traumatic events were not assessed, which could have impacted the wellbeing of postpartum mothers [35,36]. Fifth, this study would have benefitted from the inclusion of a passive control group. Sixth, seven participants stopped the surprise mental arithmetic task before entirely completing it, leading to missing HRV data. To our knowledge, previous studies did not report such details. Consequently, we cannot compare whether this early stress phase termination is higher in our sample than in other populations. Finally, although the screening questions were based on PTSD stressor criteria of DSM-5 and previously used [1,18,48,63], assessing CB-PTSD risk with only these two items is an important limitation, and validated assessment of CB-PTSD risk should be developed to be considered in future studies.

### 4.2. Future Research Perspectives

The literature suggests that an altered stress activity might play a major role in the development of PTSD and, by extension, CB-PTSD [4,5]. In our study, high-risk mothers perceived higher stress levels and showed altered ANS and HPA activation during the LICSP stress paradigm, when controlling for the perceived infant life threat. Given that important research gaps persist, further studies need to be conducted to better understand these psychological and physiological stress mechanisms at such an early time period after giving birth and how these are linked to the development of CB-PTSD later on. The findings of this study need replication in a larger sample, and the LICSP procedure would benefit from further adaptations, including changing the conditions for the baseline assessment and improving the psychosocial stressor of the LICSP in order to elicit more intense physiological stress responses. Adding a passive control group is needed to better understand the LICSP’s potential in eliciting physiological stress responses. Regarding the utility of the LICSP to identify biomarkers specific to CB-PTSD, longitudinal studies need to be conducted, with CB-PTSD assessment at least one month following childbirth.

## Figures and Tables

**Table 1:** Descriptive characteristics of the psychophysiological stress assessments.

	Total Sample (*n* = 52)	Low-Risk Group (*n* = 28)	High-Risk Group (*n* = 24)
	*M SD*	*M SD*	*M SD*
**Salivary cortisol (nmol/L)**			
C1 level	5.5 (2.57)	5.31 (2.57)	5.72 (2.6)
C2 level	4.96 (2.19)	4.87 (2.27)	5.06 (2.15)
C3 level ^1^	4.98 (2.13)	5.03 (2.31)	4.92 (1.96)
C4 level ^1^	5.07 (2.35)	5.26 (2.79)	4.86 (1.79)
C5 level ^1^	5.12 (2.47)	5.13 (2.8)	5.12 (2.12)
C6 level ^1^	4.7 (2.13)	4.79 (2.29)	4.61 (1.98)
C7 level ^1^	4.51 (2.19)	4.65 (2.51)	4.35 (1.82)
**HF power (ms^2^)**			
HF1	359.56 (472.95)	409.07 (593.5)	301.79 (275.74)
HF2 ^2^	386.04 (364.14)	371.93 (363.87)	401.92 (371.61)
HF3 ^23^	454.3 (417.7)	413.04 (421.67)	499.48 (418.85)
HF4 ^1^	369.2 (427.57)	416.19 (508.43)	318.29 (321.41)
**LF power (ms^2^)**			
LF1	646.08 (572.69)	609.82 (574.26)	688.38 (580.22)
LF2 ^2^	497.08 (440.61)	410.44 (280.52)	594.54 (560.67)
LF3 ^23^	1299.73 (1101.02)	1372.04 (1336.12)	1220.52 (793.71)
LF4^1^	663.36 (483.53)	602.81 (491.58)	728.96 (476.23)
**LF/HF ratio**			
LF/HF1	2.95 (2.22)	2.67 (1.81)	3.27 (2.62)
LF/HF2 ^2^	2.17 (3.09)	2.26 (3.96)	2.06 (1.72)
LF/HF3 ^23^	3.74 (2.79)	4.39 (3.18)	3.03 (2.13)
LF/HF4 ^1^	2.71 (1.78)	2.38 (1.53)	3.07 (1.98)
**Perceived stress**			
VAS1	1.67 (0.83)	1.54 (0.79)	1.83 (0.87)
VAS2	1.54 (0.67)	1.5 (0.64)	1.58 (0.72)
VAS3	2.43 (0.96)	2.25 (0.7)	2.65 (1.18)
VAS4	2.85 (1.19)	2.89 (1.17)	2.79 (1.25)
VAS5 ^1^	3.38 (1.29)	3.38 (1.24)	3.38 (1.38)
VAS6 ^1^	2.24 (1.19)	1.96 (1.04)	2.54 (1.28)
VAS7 ^1^	1.68 (0.87)	1.5 (0.81)	1.88 (0.9)
VAS8 ^1^	1.56 (0.73)	1.38 (0.57)	1.75 (0.85)
VAS9 ^1^	1.46 (0.71)	1.23 (0.51)	1.71 (0.81)
VAS10 ^1^	1.27 (0.53)	1.15 (0.46)	1.39 (0.57)

Note. C1 = 1st salivary cortisol sample during the baseline; C2 = 2nd salivary cortisol sample taken before the psychosocial and cognitive stressors; C3 = 3rd salivary cortisol sample during recovery after the stress task; C4 = 4th salivary cortisol sample during early recovery; C5 = 5th salivary cortisol sample during mid recovery; C6 = 6th salivary cortisol sample during late recovery; C7 = 7th salivary cortisol sample during the recovery at the end of the paradigm; HF = high-frequency power; HF1 = HF during baseline; HF2 = HF during the psychosocial stressor; HF3 = HF during the cognitive stressor; HF4 = HF during the recovery; LF = low-frequency power; LF1 = LF during baseline; LF2 = LF during the psychosocial stressor; LF3 = LF during the cognitive stressor; LF4 = LF during the recovery; LF/HF1 = LF/HF ratio during baseline; LF/HF2 = LF/HF ratio during the psychosocial stressor; LF/HF3 = LF/HF ratio during the cognitive stressor; LF/HF4 = LF/HF ratio during the recovery; VAS = visual analog scale ranging from 1 = not all stressed to 5 = extremely stressed; VAS1 = perceived stress at the beginning of the LICSP; VAS2 = perceived stress at C1; VAS3 = perceived stress at C2; VAS4 = perceived stress at the end of the ICT; VAS5 = perceived stress at the end of the arithmetic task; VAS6 = perceived stress at C3; VAS7 = perceived stress at C4; VAS8 = perceived stress at C5; VAS9 = perceived stress at C6; VAS10 = perceived stress at C7. See Figure 1 for the detailed study procedure. ^1^ Two participants of the low-risk group dropped at the end of the stress phase, resulting in two missing data points. ^2^ Due to technical issues, the cardiac activity of one participant of the low-risk group was not recorded. Given > 30% of her data were missing, these data were not imputed. ^3^ Seven participants (*n_low-risk_* = 4, *n_high-risk_* = 3) decided to stop the surprise arithmetic task before the recording of the 3 min cardiac activity.

**Table 2:** Descriptive data of the sample.

	Total Sample (*n* = 52)	Low-Risk Group (*n* = 28)	High-Risk Group (*n* = 24)	Group Difference
**Sociodemographic and medical characteristics**				
Age (M, SD)	31.71 (4.00)	33.48 (4.00)	31.83 (3.89)	*W* = 382.00, *p* = 0.276
Missing data (N, %)	1 (1.92)	1 (3.57)	0	
Civil status				*p* = 0.507
Married or in a relationship (N, %)	36 (69.23)	20 (71.43)	16 (66.67)	
Single, separated, divorced, or widowed (N, %)	7 (13.46)	3 (10.71)	4 (16.67)	
Other (N, %)	2 (3.87)	2 (7.14)	0	
Missing data (N, %)	7 (13.46)	3 (10.71)	4 (16.67)	
Education level				*p* = 0.407
Compulsory education (N, %)	3 (5.77)	2 (7.14)	1 (4.17)	
Post-compulsory education (N, %)		1 (3.57)	2	
Apprenticeship (N, %)	6 (11.54)	2 (7.14)	4 (16.67)	
University (N, %)	30 (57.69)	17 (60.71)	13 (54.17)	
Other (N, %)	3 (5.77)	3 (10.71)	0	
Missing data (N, %)	7 (13.46)	3 (10.71)	4 (16.67)	
Smoking				*p* = 0.242
Yes (N, %)	3 (5.77)	3 (10.71)	0	
No (N, %)	42 (80.77)	22 (78.58)	20 (83.33)	
Missing data (N, %)	7 (13.46)	3 (10.71)	4 (16.67)	
Parity (M, SD)	0.40 (0.72)	0.57 (0.74)	0.21 (0.66)	*W* = 436.00, *p* = 0.022
Gravidity (M, SD)	1.65 (1.25)	1.89 (1.23)	1.38 (1.24)	*W* = 450.00, *p* = 0.013
Type of delivery				*p* < 0.001
Vaginal birth (N, %)	28 (53.85)	22 (78.57)	6 (25.00)	
Planned cesarean section (N, %)	6 (11.54)	5 (17.86)	1 (4.17)	
Vacuum-assisted vaginal delivery (N, %)	1 (1.92)	0	1 (4.17)	
Forceps delivery (N, %)	3 (5.77)	0	3 (12.50)	
Emergency cesarian section (N, %)	14 (26.92)	1 (3.57)	13 (54.17)	
**Psychological characteristics**				
Perceived life threat for the mother (M, SD)	1.77 (1.63)	1.07 (0.26)	2.58 (2.13)	*W* = 211.00, *p* = 0.002
Perceived life threat for the infant (M, SD)	3.00 (2.22)	1.21 (0.42)	5.08 (1.53)	*W* = 6.00, *p* < 0.001
Anxiety, HADS-A (M, SD)	6.91 (3.65)	6.40 (3.25)	7.58 (4.1)	*W* = 198.50, *p* = 0.360
Cronbach’s Alpha	0.72	0.68	0.76	
Missing data (N, %)	8 (15.38)	3 (10.71)	5 (20.83)	
Depression, EPDS (M, SD)	6.75 (5.13)	5.24 (3.75)	8.74 (6.06)	*W* = 155.00, *p* = 0.051
Cronbach’s Alpha	0.85	0.76	0.90	
Missing data (N, %)	7 (13.46)	3 (10.71)	5 (20.83)	

Note. HADS-A = anxiety subscale of the Hospital Anxiety and Depression Scale (range = 0–21); EPDS = Edinburgh Postnatal Depression Scale (range = 0–30). Significant group differences were tested with Fischer’s exact test (*p*) or Mann–Whitney test (W).

**Table 3:** Descriptive data of the Lausanne Infant Crying Stress Paradigm.

	Total Sample (*n* = 52)	Low-Risk Group (*n* = 28)	High-Risk Group (*n* = 24)	Group Differences
Time between birth and the LICSP (hh:mm) (M, SD)	51:04 (22:02)	46:23 (21:50)	56:31 (21:27)	*W* = 236, *p* = 0.068
LICSP start time (hh:mm p.m.) (M, SD)	1:13 (0:20)	1:13 (0:20)	1:15 (0:20)	*W* = 309.5, *p* = 0.632
Baseline phase duration (mm:ss) (M, SD)	43:43 (3:28)	42:58 (2:45)	44:35 (4:01)	*W* = 256.5, *p* = 0.146
Stress phase duration (mm:ss) (M, SD)	21:41 (2:39)	21:58 (3:00)	21:23 (2:01)	*W* = 349, *p* = 643
Missing data (N, %)	1 (1.92)	1 (3.57)	0	
Recovery phase duration (mm:ss) (M, SD)	42:37 (4:28)	43:10 (5:53)	42:02 (2:02)	*W* = 341.5, *p* = 0.561
Missing data (N, %)	2 (3.85)	2 (7.14)	0	
LICSP duration (mm:ss) (M, SD)	107.68 (4.61)	107.38 (4.73)	108 (4.56)	*W* = 289.5, *p* = 0.668
Missing data (N, %)	2 (3.85)	2 (7.14)	0	
Breastfeeding within the hour preceding the stress phase				*X*^2^(1) = 0.06, *p* = 0.799
Yes (N, %)	9 (17.31)	4 (14.29)	5 (20.83)	
No (N, %)	43 (82.69)	24 (85.71)	19 (79.17)	
**Characteristics of the stress phase**				
Novelty (M, SD)	77.80 (28.34)	72.12 (29.91)	83.96 (25.75)	*W* = 220.5, *p* = 0.061
Missing data (N, %)	2 (3.85)	2 (7.14)	0	
Difficulty (M, SD)	63.96 (22.88)	65.31 (23.42)	62.50 (22.70)	*W* = 335, *p* = 0.66
Missing data (N, %)	2 (3.85)	2 (7.14)	0	
Stress (M, SD)	50.16 (27.09)	52.81 (24.61)	47.29 (29.82)	*W* = 345, *p =* 0.525
Missing data (N, %)	2 (3.85)	2 (7.14)	0	
Controllability (M, SD)	49.12 (29.19)	54.04 (33.32)	43.79 (23.48)	*W* = 376, *p* = 0.212
Missing data (N, %)	2 (3.85)	2 (7.14)	0	
Predictability (M, SD)	32.73 (29.23)	36.88 (27.22)	28.00 (31.36)	*W* = 303, *p* = 0.247
Missing data (N, %)	7 (13.46)	4 (14.29)	3 (12.50)	

Note. LICSP = Lausanne Infant Crying Stress Paradigm. Significant group differences were tested with Mann–Whitney (W) or chi-square (*X*^2^) tests. Characteristics of the stress phase were assessed on a 100-point Likert scale, with 0 = *not at all*, 50 = *moderately*, 100 = *extremely*.

## Data Availability

The data presented in this study are available on request from the corresponding author. The data are not publicly available due to the lack of informed consent from participants for open access publication of their data.
